# Pharmacogenetics-based population pharmacokinetic analysis and dose optimization of valproic acid in Chinese southern children with epilepsy: Effect of *ABCB1* gene polymorphism

**DOI:** 10.3389/fphar.2022.1037239

**Published:** 2022-11-25

**Authors:** Xianhuan Shen, Xinyi Chen, Jieluan Lu, Qing Chen, Wenzhou Li, Jiahao Zhu, Yaodong He, Huijuan Guo, Chenshu Xu, Xiaomei Fan

**Affiliations:** ^1^ Shenzhen Baoan Women’s and Children’s Hospital, Jinan University, Shenzhen, China; ^2^ College of Pharmacy, Jinan University, Guangzhou, China; ^3^ School of Pharmaceutical Sciences, Health Science Center, Shenzhen University, Shenzhen, China

**Keywords:** valproic acid, epileptic children, population pharmacokinetics, NONMEM, genetic polymorphism

## Abstract

**Objective:** The aim of this study was to establish a population pharmacokinetic (PPK) model of valproic acid (VPA) in pediatric patients with epilepsy in southern China, and provide guidance for individualized medication of VPA therapy.

**Methods:** A total of 376 VPA steady-state trough concentrations were collected from 103 epileptic pediatric patients. The PPK parameter values for VPA were calculated by using the nonlinear mixed-effects modeling (NONMEM) method, and a one-compartment model with first-order absorption and elimination processes was applied. Covariates included demographic information, concomitant medications and selected gene polymorphisms. Goodness-of-fit (GOF), bootstrap analysis, and visual predictive check (VPC) were used for model evaluation. In addition, we used Monte Carlo simulations to propose dose recommendations for different subgroup patients.

**Results:** A significant effect of the patient age and *ABCB1* genotypes was observed on the VPA oral clearance (CL/F) in the final PPK model. Compared with patients with the *ABCB1* rs3789243 AA genotype, CL/F in patients with GG and AG genotypes was increased by 8% and reduced by 4.7%, respectively. The GOF plots indicated the satisfactory predictive performance of the final model, and the evaluation by bootstrap and VPC showed that a stable model had been developed. A table of individualized dosing regimens involving age and *ABCB1* genotype was constructed based on the final PPK model.

**Conclusion:** This study quantitatively investigated the effects of patient age and *ABCB1* rs3789243 variants on the pharmacokinetic variability of VPA. The PPK models could be beneficial to individual dose optimization in epileptic children on VPA therapy.

## 1 Introduction

Epilepsy is one of the most common and most disabling chronic neurological disorders, characterized by an enduring predisposition to generate recurrent epileptic seizures (Devinsky et al., 2018). There are more than 70 million people of all age groups worldwide who suffer from epilepsy (Löscher et al., 2020). The prevalence of epilepsy in children is particularly high, ranging from 4 to 9 per 1,000 children, and is increasing each year (Mac et al., 2007). Valproic acid (VPA) is currently recommended by the ‘International League against Epilepsy’ (ILAE) as a first-line antiepileptic drug (AED) for children (Glauser et al., 2013), due to its broad spectrum of action against various kinds of seizures.

The exact mechanisms of VPA remain to be understood. Nevertheless, several studies have proposed that VPA can intensify the synthesis and release of gamma aminobutyric acid (GABA), an inhibitory neurotransmitter in the central nervous system, thereby suppress seizures (Rogawski and Löscher, 2004; Berg et al., 2008). VPA is rapidly and completely absorbed, and the available data have suggested the nearly total bioavailability (close to 1.0) for oral solutions and capsules (Johannessen and Johannessen, 2003). It is a highly protein-bound drug to albumin, and its protein binding is concentration-dependent (Patsalos et al., 2017). The saturable binding and a higher unbound fraction exhibit when VPA concentrations above 50 mg/L, which results in a non-linear relationship between daily dose and serum concentration (Perucca, 2002; Patsalos et al., 2008). For VPA half-life, an average value is 10–12 h. Additionally, higher VPA clearance and shorter half-life have been reported in children (Methaneethorn, 2018).

VPA is mainly metabolized by the liver, with only a small amount of unchanged form being excreted by the urine (Johannessen and Johannessen, 2003). It includes the following main routes: Glucuronidation *via* uridine diphosphate glucuronosyltransferase (UGT), beta-oxidation in mitochondria (both as major metabolic routes accounting for 50% and 40%, respectively), and a minor route of cytochrome P450 (CYP)-mediated oxidation and hydroxylation (approximately 10%) (Xu et al., 2018).

According to the Consensus Guidelines, the effective VPA therapeutic reference range for epilepsy is 50–100 mg/L with a broad recommended dose range (Hiemke et al., 2018). Some researchers have reported that the rates of adverse reactions (including nausea, vomiting, weight gain, teratogenicity and hepatotoxicity) were higher in patients with VPA levels >125 mg/L (Zang et al., 2022). However, serum concentration levels may vary considerably among patients taking the same dose of VPA (Ferraro and Buono, 2005). Several factors involved in VPA absorption (diet and dosage form), distribution (body weight, age, dose, and protein binding), and metabolism (sex, dose, gene polymorphism of enzymes related to VPA glucuronidation and oxidation, drug-drug interactions with other commonly used AEDs) have a significant influence on VPA clearance, which cause to the high inter- and intra-individual variability of VPA pharmacokinetic (PK) (Mei et al., 2018). The large variability in VPA and narrow therapeutic window necessitate therapeutic drug monitoring (TDM) and individualized dosing regimens, thus ensuring optimal efficacy and avoiding adverse effect, especially in pediatric patients.

A population pharmacokinetic (PPK) modeling approach can help identify the quantitative impact of individual variability on VPA PK in a target population (Kiang et al., 2012). Compared with traditional empirical dosing, model-informed precision dosing (MIPD) can aid in optimizing individual dosing based on patient physiology, pathology, genetics and other characteristics to improve the attainment of the predefined targets (Darwich et al., 2021). In addition, this new approach is more flexible in clinical applications such as non-steady state drug concentrations or clinically unstable patients (Methaneethorn, 2018). Analysis of PPK requires sparse PK sampling from patients and is applicable to children in particular. To date, although there have been a substantial number of PPK studies in the pediatric population addressing the association of demographic factors with PK variability of VPA (Botha et al., 1995; Correa et al., 2008; Ding et al., 2015; Rodrigues et al., 2018), few of them were focused on the effect of genetic polymorphisms (Jiang et al., 2007; Xu et al., 2018; Guo et al., 2020).

With the development of the pharmacogenomics in VPA, it has helped to identify a large number of candidate genes, such as drug metabolizing enzymes, regulating signaling pathways (membrane transporters and nuclear receptors), effect pathways related gene mutations, associating with the increase or decrease of VPA serum concentration (Ghodke-Puranik et al., 2013). Besides, genes that have been demonstrated in influencing its PK behavior could also partly illustrate inter-individual variability among patients taking VPA, including *UGT1A3/1A4/1A6/1A8/1A9/1A10/2B7*, *CYP2A6/2B6/2C9/2C19*, leptin receptor (*LEPR*), ABC transporter, adenosine monophosphate-activated protein kinase (*AMPK*), and sodium channel neuronal type I alpha subunit (*SCN1A*) (Zhu et al., 2017; Xu et al., 2018). However, little is known about the quantitative impact of these genotypes as covariates on variability in the PK parameters of VPA in children with epilepsy.

The purpose of this study is to identify potential covariates (including clinical and genetic factors) that could explain the PK variability of VPA within the Chinese pediatric population, and to establish mathematical modeling reflecting these covariates using the nonlinear mixed-effects modeling (NONMEM) method. It is expected that this PPK model can provide information in the clinic for the individualization of VPA dosage in epileptic children.

## 2 Materials and methods

### 2.1 Patients and data collection

All the patients were diagnosed with definitely characterized epilepsy or epileptic syndrome by two independent neurologic clinicians on the bias of the latest version of ILAE commission’s classification criterion (Scheffer et al., 2017). Serum samples for VPA trough concentration determination at a steady-state were collected retrospectively from pediatric patients with epilepsy in Baoan Women’s and Children’s Hospital (Shenzhen, China) from September 2016 to January 2022. VPA was administered orally two to three times a day in the forms of syrup (Depakine, Hangzhou Sanofi Minsheng Pharmaceutical Co. Ltd., Hangzhou, China) or conventional tablets (Hunan Xiangzhong Pharmaceutical Co. Ltd., Hunan, China).

The children with epilepsy aged <16 years old who received VPA alone or in combination with other AEDs were included in this study. Those with a history of pseudo-epileptic seizure, impaired hepatic and/or renal function, or the existence of any diseases which presented gastrointestinal symptoms similar to side effects induced by antiepileptic drugs were excluded. Comprehensive demographic information was collected for the patients at the time of enrollment in the study, including age, weight, sex, VPA dosage regimen details (dose, dosing time, and frequency), VPA total serum concentrations and concurrent medications.

The study was approved by the Baoan Women’s and Children’s Hospital Ethics Committee (Appr. Number LLSC 2020-10-06-KS, date of approval: 25 September 2020) and performed in accordance with the Declaration of Helsinki and its amendments. Written informed consents were obtained from all patients’ guardians.

### 2.2 Analysis of VPA in serum samples

After at least 1 week of VPA stable dosing regimens, the patients were assumed to have reached steady-state serum concentrations. Given the reported diurnal variation in VPA concentrations, and trough concentration in the morning is the most stable level, so the blood samples were obtained before the morning dose (Methaneethorn, 2018). Serum concentrations of VPA were analyzed by homogeneous enzyme immune assays (Viva-E, Siemens, Erlangen, Germany; commissioned to Kingmed Diagnostics Group Co., Ltd.). The coefficients of variation within and between assays were less than 10%, and the analytic measurement range was 1–600 mg/L with 1 mg/L as the lower limit of quantification.

### 2.3 Genotype identification

Genomic DNA samples were extracted from 1.5 ml of whole blood. Target gene fragments of single nucleotide polymorphisms (SNPs) were amplified by the polymerase chain reaction (PCR), which was performed as detailed in our previous study (Fan et al., 2020). The following SNPs were selected for genotyping by reviewing the pharmacogenetic studies related to ABC transporters, VPA metabolism, nuclear receptors, and the efficacy of VPA treatment (Kwan et al., 2009; El-Khodary et al., 2012; Nakashima et al., 2015; Queckenberg et al., 2015; Li et al., 2016; Talwar et al., 2017; Chen et al., 2018; Margari et al., 2018; Wang et al., 2018; Xu et al., 2018; Al-Eitan et al., 2019; Chouchi et al., 2019; Shi et al., 2019; Liu et al., 2020; Makowska et al., 2021). Genotyping of all polymorphisms was carried out using Sequenom MassArray System (Agena Bioscience, San Diego, CA, United States) and iPLEX^®^ Gold Assay. The MassArray Typer 4.0 software was used for data acquisition and analysis. All SNPs were calculated to confirm if they were in Hardy–Weinberg equilibrium.

### 2.4 Population pharmacokinetic model development

The PPK model of VPA for pediatric patients was established using non-linear mixed effect modeling software, NONMEM^®^ program (version 7.5, ICON Development Solutions, Ellicott City, MD, United States), to describe the relationship between VPA serum concentrations and time data and to conduct model-based simulations. The output visualizations and the model evaluations were performed in the R programming environment (version 4.1, http://www.r-project.org) and Pirana^®^ (version 3.0, http://www.pirana-software.com). The first-order conditional estimation method with interaction (FOCE-I) was used to estimate the PK pharmacokinetic parameters and their variability.

#### 2.4.1 Base model

Based on previous reports, the VPA concentration-time data were fitted by a one-compartment model with first-order absorption and elimination (Ding et al., 2015). NONMEM subroutines were specified as ADVAN2-TRANS2. Since the majority of the data collected were trough concentration measurements, there is no information to identify the absorption rate constant (K_a_). A previous study has shown that the k_a_ had no significant impact on clearance estimates (Byon et al., 2013). Therefore, K_a_ was fixed at 1.9 h^−1^, in accordance with the references (Ding et al., 2015). Moreover, as bioavailability could not be determined either, clearance (CL) and the volume of distribution (V) were regarded as the apparent clearance (CL/F) and apparent distribution volume (V/F), respectively. The inter-individual variability was evaluated on PK parameters using an exponential model. Additive, proportional and combined error models were investigated to describe the residual variability.

#### 2.4.2 Covariate model

The covariate models were developed using a stepwise forward/backward approach. After the construction of the base model, the continuous covariates (AGE, WT, VPA daily dose) and categorical covariates (SEX, concurrent medications and genotype) were used to establish a stepwise full regression model in the form of linear, power, exponential and piecewise model. As most concentrations collected in this study were trough concentrations, the covariates were investigated only for the CL/F. Covariates included in the model were first identified using graphical methods. Co-medications administered with a proportion of more than 2.5% of the sample were evaluated including levetiracetam (LEV), oxcarbazepine (OXC), topiramate (TPM), clonazepam (CNZ), phenobarbital (PB), and midazolam (MDZL). Polymorphisms of 15 candidate genes were tested for their impacts on VPA CL/F by categorizing patients into genotypic groups (wild type, heterozygous mutation, and homozygous mutation).

During the process of stepwise forward inclusion and backward elimination, criterion for the selection of a model was when the objective function value (OFV) changed at least 3.84 units (∆OFV >3.84) between the two nested models (*p* < 0.05, df = 1), and the covariate was considered to have a significant effect. The differences in OFV, Akaike Information Criterion (AIC), Bayesian Information Criterion (BIC), and parameter estimates rationality of each model were comprehensively compared to select the optimal model. Models with lower AICs and BICs were considered superior. This process was continued until no further change in the OFV was observed (Byon et al., 2013).

### 2.5 Model evaluation

The appropriateness and stability between the base model and the final model was first evaluated by visual inspection of goodness-of-fit (GOF) plots. GOF diagnostic scatter plots are as follows: observed (DV) vs. predicted concentrations, conditional weighted residual errors (CWRES) vs. time or predicted concentrations. In addition, visual predictive checks (VPC) with 1,000 simulation data sets was performed for the predictive performance of final model, and the 5th, 50th, and 95th percentile of the observations and 95% confidence interval (95% CI) of simulated concentrations were plotted verse time. Furthermore, a bootstrap resampling method (1,000 runs) was applied to calculate the median and 95% CI of parameters, and these values were then compared with the estimated values obtained from the final model so as to assess the robustness and accuracy simultaneously.

### 2.6 Simulation of dosing regimen

Probability of target attainment (PTA) table was generated by performing Monte Carlo simulations (*n* = 10,000) using the final model. The steady-state trough concentration of VPA was investigated and the goal was to have VPA concentrations within 50–100 mg/L. Dosing regimens at 15–35 mg/kg/day every 12 h administered orally were considered for determination of initial therapeutic VPA dose. Virtual patients were divided into different subgroups on the basis of the incorporated covariates, and PTA at least 70% probability was considered to be clinically acceptable. In children, VPA dose was mainly depended on the body weight. Therefore, WT of 10–40 kg was constructed for typical pediatric patients based on the China National Survey of Body Weight for children (Li et al., 2009), as we had few patients >40 kg in our cohort (*n* = 8). Simulations were conducted for different WT subgroups to determine the most appropriate scheme to meet the therapeutic criteria. The dose regimens were then compared with those from other PPK studies.

## 3 Results

### 3.1 Patient demographic data and genotyping

A total of 376 steady-state trough concentrations (range, 14.67–110.99 mg/L) obtained from 103 pediatric patients (47 females and 56 males) with epilepsy were included in the final analysis. Table 1 shows the main demographic characteristics of the patients along with concomitant medications. VPA was administered orally two to three times daily, and co-prescribed medications in this population mainly included levetiracetam and oxcarbazepine. Genetic testing was also performed for all 103 patients. The genotype with allele frequencies is illustrated in Table 2. The deviations from Hardy-Weinberg equilibriums for the selected SNPs were assessed using the chi-square test, and 12 genotypes were found to conform to the equilibrium (*p* > 0.05), while *CYP2C9* rs1057910, *MTHFR* rs1801133, *SCN1A* rs6732655 significantly deviated from Hardy–Weinberg proportions (*p* < 0.05) and were excluded in the covariate model development.

**TABLE 1:** A summary of demographic information in children with epilepsy.

Characteristics	Values
Number of patients/VPA samplings	103/376
Male/Female	56/47
Age (years)	5.30 ± 3.39 (0.5–15)
Weight (kg)	19.9 ± 10.6 (6.5–52.0)
VPA daily dose (mg/kg/day)	23.8 ± 5.7 (9.9–45.7)
VPA concentration (mg/L)	60.54 ± 19.32 (14.67–110.99)
Co-medicated drugs, *n* (%)	
Levetiracetam	52 (13.83%)
Oxcarbazepine	42 (11.17%)
Topiramate	16 (4.26%)
Clonazepam	14 (3.72%)
Phenobarbital	12 (3.19%)
Midazolam	10 (2.66%)
Ibuprofen	6 (1.6%)

**TABLE 2:** Genotype frequencies of selected variants in 103 patients.

Genetic polymorphisms	Genotypes	Values(*n*)	Frequency(%)	HWE(*P*-value)
*ABCB1*				
rs3789243	AA/AG/GG	14/50/39	13%/49%/38%	0.748
rs1128503	AA/AG/GG	42/51/10	41%/50%/9%	0.329
*ABCC2*				
rs2273697	GG/GA/AA	88/14/1	85%/14%/1%	0.603
*CYP1A1*				
rs2606345	CC/CA/AA	96/7/0	93%/7%/0%	0.721
*CYP2C9*				
rs1057910	AA/AC/CC	96/6/1	93%/6%/1%	0.026
*LEPR*				
rs1137101	AA/AG/GG	2/19/82	2%/18%/80%	0.477
*MTHFR*				
rs1801133	GG/GA/AA	51/35/17	50%/34%/16%	0.016
rs1801131	TT/TG/GG	67/31/5	65%/30%/5%	0.57
*SCN1A*				
rs6732655	AA/AT/TT	4/97/2	4%/94%/2%	0
rs6730344	CC/CA/AA	74/26/3	72%/25%/3%	0.699
rs10167228	TT/TA/AA	1/17/85	1%/16%/83%	0.884
rs3812718	CC/CT/TT	17/47/39	16%/46%/38%	0.657
rs2298771	CC/CT/TT	1/18/84	1%/17%/82%	0.974
*SCN2A*				
rs2304016	AA/AG/GG	82/20/1	80%/19%/1%	0.857
rs17183814	GG/GA/AA	73/28/2	71%/27%/2%	0.715

*p* > 0.05 represents that the distribution of genotype follows Hardy-Weinberg equilibrium.

### 3.2 Population pharmacokinetic model development

An additive error model describing residual variability with the lowest AIC and BIC was selected, according to the distribution of residuals in the diagnostic plots of the base model. As only steady-state trough serum concentrations were collected, the relative standard error (RSE) of the inter-individual variability (IIV) for V/F was poor and then removed from the model building process. Furthermore, none of the tested covariates could significantly influence V/F. The population typical value of CL/F and V/F in the base model was 0.205 L/h and 3.43 L, respectively.

The changes of OFV value in the covariate screening process are listed in Table 3. Only 1.6% of the population had concomitant administration of ibuprofen and thus was not included as a covariate. A total of 12 SNPs were selected to test the impact of genetic factors on the CL/F of VPA in children with epilepsy. In the first forward inclusion, clonazepam decreased the OFV by 3.920 (*p* < 0.05). *ABCB1* rs1128503 and *SCN1A* rs3812718 dropped the OFV value from 2595.456 to 2589.456 and 2587.310, respectively (*p* < 0.05). However, there was no significant difference for the above three covariates in the second inclusion. Age and *ABCB1* rs3789243 were identified as significant covariates on the CL/F and retained in the final model followed by forward inclusion and backward elimination. Further incorporation of other covariates did not improve the fitting performance of the model to the observed data.

**TABLE 3:** Results of hypothesis testing in the model development procedure.

Model no.	Model description	OFV	ΔOFV	*P*-value
Forward inclusion 1				
1	Base model	2595.456		
2	Add WT on CL/F in Model 1	2435.963	−159.493	<0.001
3	Add AGE on CL/F in Model 1	2417.706	−177.750	<0.001
4	Add TDD on CL/F in Model 1	2595.416	−0.040	NS
5	Add SEX on CL/F in Model 1	2595.347	−0.109	NS
6	Add LEV on CL/F in Model 1	2595.468	0.012	NS
7	Add OXC on CL/F in Model 1	2593.070	−2.386	NS
8	Add TPM on CL/F in Model 1	2593.728	−1.728	NS
9	Add CNZ on CL/F in Model 1	2591.536	−3.920	<0.05
10	Add PB on CL/F in Model 1	2595.455	−0.001	NS
11	Add MDZL on CL/F in Model 1	2591.975	−3.481	NS
12	Add *ABCB1* rs3789243 on CL/F in Model 1	2586.368	−9.088	<0.01
13	Add *ABCB1* rs1128503 on CL/F in Model 1	2589.456	−6.000	<0.05
14	Add *ABCC2* rs2273697 on CL/F in Model 1	2594.987	−0.469	NS
15	Add *CYP1A1* rs2606345 on CL/F in Model 1	2594.706	−0.750	NS
16	Add *LEPR* rs1137101 on CL/F in Model 1	2592.391	−3.065	NS
17	Add *MTHFR* rs1801131 on CL/F in Model 1	2595.074	−0.382	NS
18	Add *SCN1A* rs6730344 on CL/F in Model 1	2593.524	−1.932	NS
19	Add *SCN1A* rs10167228 on CL/F in Model 1	2594.216	−1.240	NS
20	Add *SCN1A* rs3812718 on CL/F in Model 1	2587.310	−8.146	<0.01
21	Add *SCN1A* rs2298771 on CL/F in Model 1	2593.886	−1.570	NS
22	Add *SCN2A* rs2304016 on CL/F in Model 1	2592.423	−3.033	NS
23	Add *SCN2A* rs17183814 on CL/F in Model 1	2594.704	−0.752	NS
Forward inclusion 2				
24	Add CNZ on CL/F in Model 3	2413.940	−3.766	NS
25	Add *ABCB1* rs3789243 on CL/F in Model 3	2408.473	−9.233	<0.01
26	Add *ABCB1* rs1128503 on CL/F in Model 3	2415.917	−1.789	NS
27	Add *SCN1A* rs3812718 on CL/F in Model 3	2415.719	−1.987	NS
Backward elimination				
28	Remove AGE on CL/F from Model 25	2586.368	177.895	<0.001
29	Remove *ABCB1* rs3789243 on CL/F from Model 25	2417.706	9.233	<0.01

WT, weight; TDD, VPA total daily dose; LEV, levetiracetam; OXC, oxcarbazepine; TPM, topiramate; CNZ, clonazepam; PB, phenobarbital; MDZL, midazolam.

Minimization and the covariance step were successful for the final model. Table 4 lists the estimate, RSE, IIV, and residual variability (RV) of the parameters for the base model, final model, and bootstrap validation. These estimates demonstrated an acceptable precision (RSE% < 30%). In the final model, the IIV-shrinkage and RV-shrinkage was 13.0% and 5.9%, respectively. The typical value of CL/F and V/F was 0.214 L/h and 3.63 L, respectively. The final model was listed below:

$$CL/F={0.214\times \left(\frac{Age}{5}\right)}^{0.357}\times 0.953^{ABCB1\;AG}\times 1.08^{ABCB1\;GG}$$ (1)

V/F=3.63 (2)

Where *ABCB1* is the *ABCB1* rs3789243 polymorphism, *ABCB1* AG = 0, GG = 0 for patients with wild type, *ABCB1* AG = 1, GG = 0 for patients with heterozygous AG genotype, *ABCB1* GG = 1, AG = 0 for patients with homozygous GG genotype.

**TABLE 4:** PPK parameter estimates from the final model and bootstrap validation.

Description	Parameter	Base model		Final model		Bootstrap^a^		Relative error (%)^b^
		Estimate	RSE (%)	Estimate	RSE (%)	Median	95% CI		
CL/F (L/h)	θ_CL_	0.205	7.4	0.214	7.4	0.207	0.136–0.245	−3.3
V/F (L)	θ_V_	3.43	22.7	3.63	23.8	3.57	1.07–6.14	−1.7
Age on CL/F	θ_Age_	—	—	0.357	9.6	0.349	0.243–0.423	−2.2
*ABCB1* rs3789243 AG on CL/F	θ_ABCB1 AG_	—	—	0.953	4.7	0.955	0.872–1.06	0.2
*ABCB1* rs3789243 GG on CL/F	θ_ABCB1 GG_	—	—	1.08	5.5	1.08	0.967–1.22	0
IIV on CL/F (%)	ω_CL_	0.302	10.9	0.169	13.0	0.167	0.106–0.42	−1.2
η-shrinkage (%)	η_shrinkage_	5.8	—	12.1	—	—	—	—
RV (mg/L)	σ_additive_	13.7	6.5	11.9	5.9	11.8	11.3–15.0	−0.8
ε-shrinkage (%)	ε_shrinkage_	12.9	—	11.1	—	—	—	—

CL/F, clearance; V/F, distribution volume; IIV, inter-individual variability; RV, residual variability; θ, factor describing the relationship between the covariate and the clearance; ω, coefficient variation of inter-individual variability; σ, coefficient variation of residual variability; RSE(%), relative standard error (standard error/estimate × 100%); 95%CI, 95% confidence interval.

^a^ 995 of 1,000 bootstrap runs were successful and used to calculate the point estimates and 95%CI.

^b^ Relative error % = (Bootstrap median − estimate in final model)/estimate in final model × 100%.

### 3.3 Model evaluation

**FIGURE 1:**
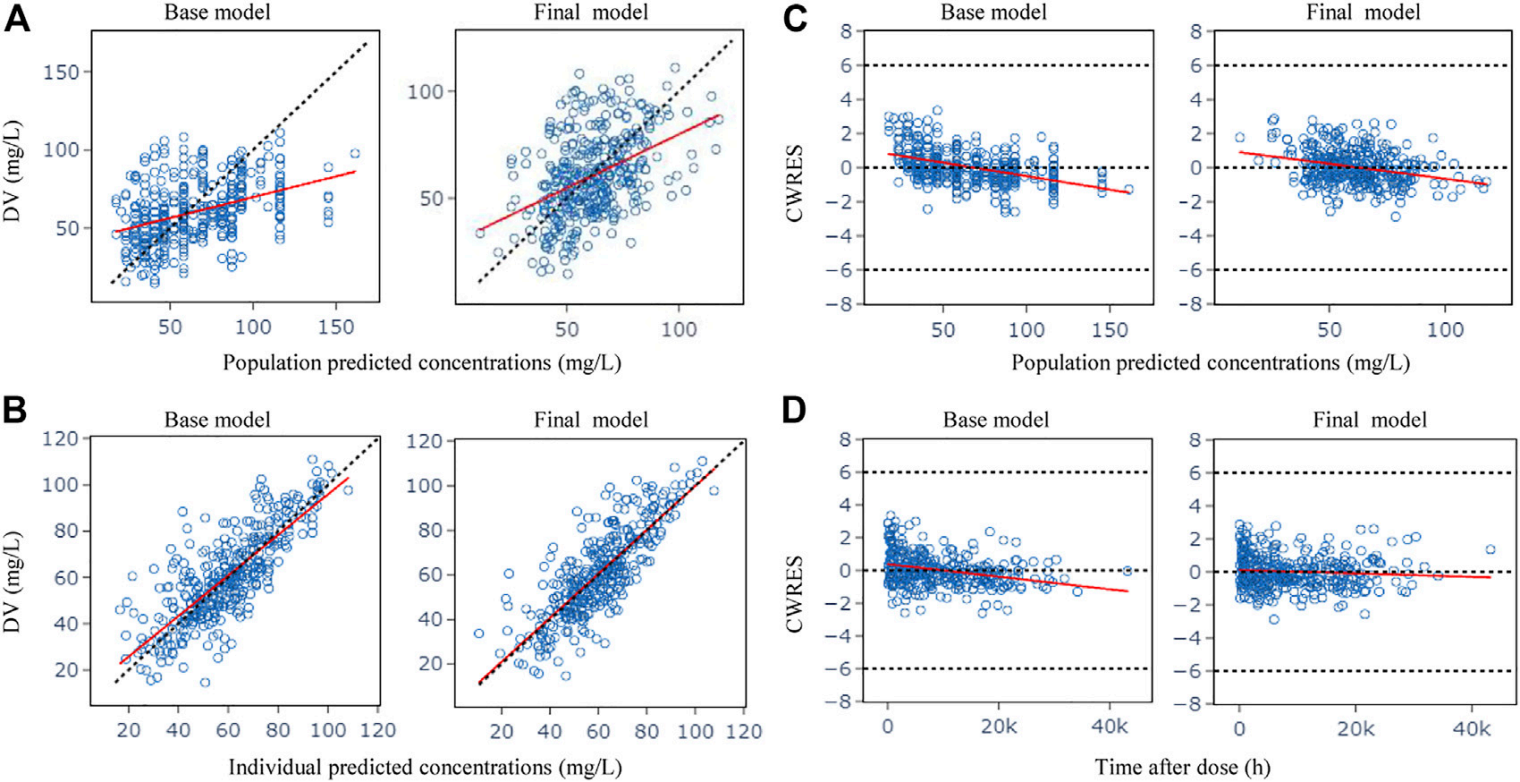
Goodness-of-fit plots for the base model and the final model. **(A)** Observed (DV) vs. population predicted concentrations (PRED); **(B)** DV vs. individual predicted concentrations (IPRED); **(C)** Conditional weighted residuals (CWRES) vs. PRED; **(D)** CWRES vs. time after dose. The red lines in the panel represent linear fit lines.

Figure 1 shows the GOF plots obtained from the base and final model. The PRED and IPRED of the final model were evenly distributed around the reference line when plotted *versus* observed concentrations, this improved correlation indicated no structural bias and a good fit of the final model prediction. The CWRES showed a distribution around 0 randomly within ±2, and the final model showed no obvious bias or significant trends compared to the base model.

The success rate (successful in minimization) of bootstrap analyses was a satisfactory result of 99.5%. The parameters observed in the final PK model were within the corresponding bootstrap 95% CI results and approximated to the median values from bootstrap with a relative error less than 3.3%, which indicated the accuracy and robustness of the final model parameter estimates (Table 4).

**FIGURE 2:**
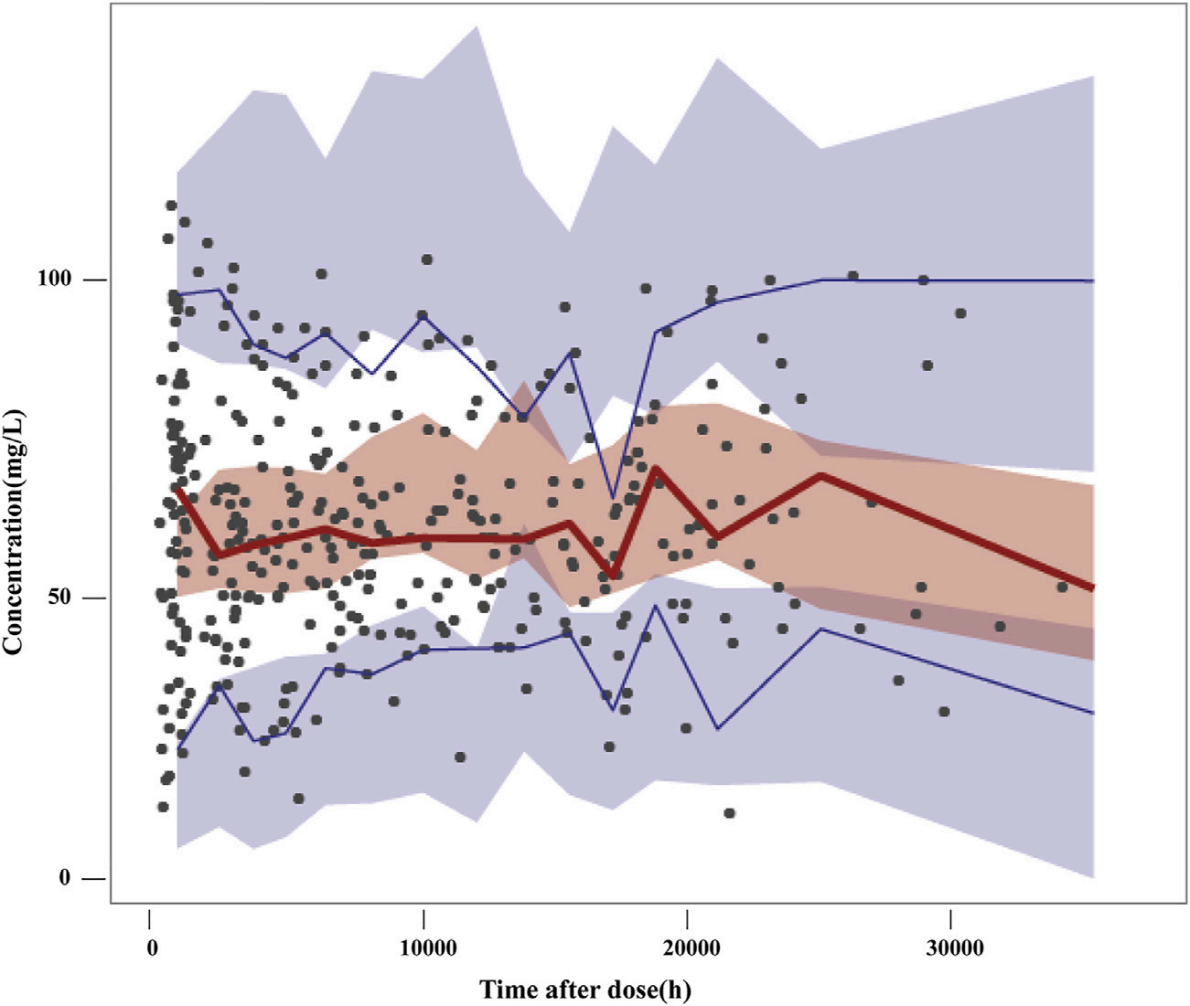
Visual predictive checks of the final model. Black dots represent the observed concentrations; the red line represents the 50th percentile of the observations; the blue lines represent the 5th and 95th percentiles of the observations. The shaded areas represent the simulation-based 95% confidence interval for each line.

The VPC of the final model is presented in Figure 2. The 5th, 50th and 95th percentiles of the observations were distributed approximately within the 95%CI of the simulated concentrations for each interval. Small outlier areas in 95th percentiles slightly over-predicted had limited impact on the overall predictive ability of the model. This suggested the presence of precise predictive performance in the final model which was judged suitable to predict VPA concentrations.

### 3.4 Simulation of dosing regimens

The PPK parameters from the final model with two covariates (age, genotype) were used to conduct Monte Carlo simulations with the goal of obtaining VPA trough concentration within 50–100 mg/L during therapy. Patients were, therefore, classified by age level and *ABCB1* genotype. Table 5 shows the simulation results of different dosing regimens. The results suggested that in children aged 1–4 years, a dose of at least 25 mg/kg/day VPA is required to achieve the PTA >70% target. For children older than 4 years, a smaller dose (around 20 mg/kg/day) is sufficient. In patients with the *ABCB1* rs3789243 homozygous (GG genotype) and heterozygous (AG genotype) types of the variant allele, CL/F was increased by 8% and reduced by 4.7%, respectively, compared with the AA genotype. Therefore, a higher dose was needed for patients with homozygous GG genotype within the same age. Taking typical patients aged 2–3 years as an example, the recommended VPA daily dose for a child with GG genotype is 30 mg/kg/day, and 25 mg/kg/day for other genotypes.

**TABLE 5:** Probability of target attainment for various predicted valproic acid daily dose using the final model.

Dose (mg/kg/day)		AGE (year)						
		1–2 (%)	2–3 (%)	3–4 (%)	4–6 (%)	6–8 (%)	8–10 (%)	10–12 (%)
*ABCB1* rs3789243 AG	15	21.86	26.17	32.77	45.32	59.71	**70.36**	**73.89**
	20	**78.80**	**76.77**	**77.75**	**83.3**	**83.23**	**71.60**	55.89
	25	**96.58**	**97.99**	**96.56**	**81.98**	63.25	48.59	33.29
	30	**78.14**	**73.83**	67.23	54.68	40.29	29.63	15.26
	35	45.17	45.84	42.79	33.68	22.53	13.41	3.95
*ABCB1* rs3789243 AA	15	14.76	17.61	26.17	38.00	53.41	64.82	**70.56**
	20	**70.11**	67.92	69.04	**74.77**	**80.24**	**72.57**	60.44
	25	**95.36**	**96.13**	**96.16**	**83.45**	67.62	54.08	39.74
	30	**85.22**	**82.39**	**73.83**	61.98	46.59	35.18	22.22
	35	56.36	54.47	49.82	42.25	29.24	19.25	8.60
*ABCB1* rs3789243 GG	15	6.44	7.88	14.43	28.41	43.55	55.60	65.66
	20	55.96	54.69	57.69	64.54	**72.46**	**70.36**	60.70
	25	**89.00**	**87.44**	**86.43**	**83.22**	**72.36**	60.36	47.03
	30	**92.7**	**91.48**	**85.21**	**70.47**	56.32	44.40	30.92
	35	**70.53**	66.16	60.82	51.73	39.96	29.67	17.57

Percentage represents the simulated patients who achieve target steady-state trough concentrations (50–100 mg/L) given the dose mentioned. Numbers in bold font indicate the PTA is greater than 70%.

**FIGURE 3:**
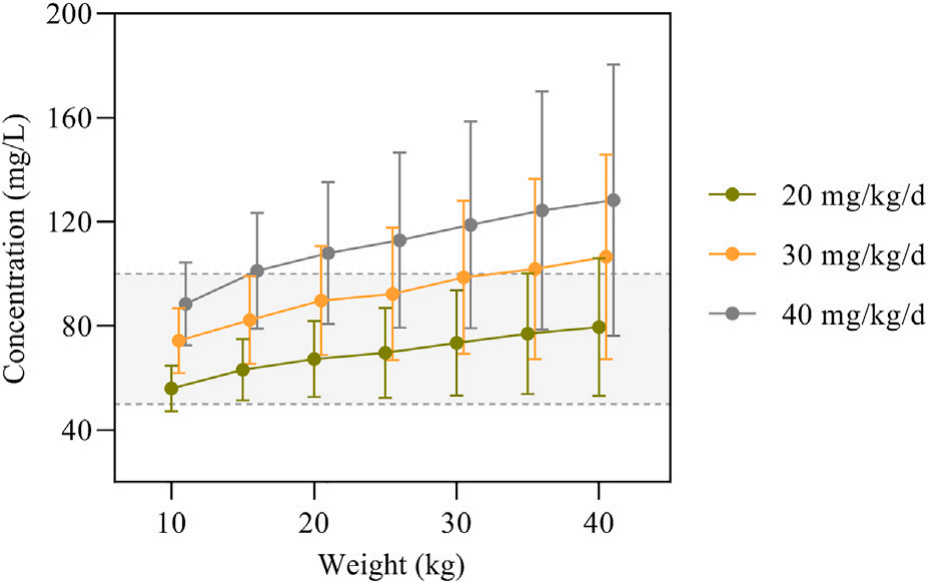
VPA trough concentrations predicted by the final model based on dose and body weight. The concentrations are shown as means with error bars representing standard deviations from the mean. The semitransparent grey field delimited by dashed lines represents the target therapeutic range (50–100 mg/L).

The simulation results of predicted VPA trough concentrations for the typical weight of 10–40 kg in children are presented in Figure 3, and the dosage regimens recommended by the other established model are displayed in Table 6. The VPA concentrations were higher with increasing body weight at the same dosage. In order to achieve the target therapeutic concentrations (50–100 mg/L), a dosing regimen of 20–30 mg/kg twice daily (bid) was required for simulated subjects with a bodyweight of 10–40 kg. Moreover, Figure 3 shows an overdose of 40 mg/kg/day in children weighing >20 kg. VPA predicted trough concentrations in excess of 100 mg/L indicate a possibly increased risk of toxicity.

**TABLE 6:** Results of doses simulations from published population pharmacokinetic models in pediatric patients with epilepsy.

Author (publication year)	Country	Target concentration (mg/L)	Weight (kg)	Recommended dose (mg/kg/day)
Yukawa et al.(1997)	Japan	75	≤10	30–40
			10–20	20–30
			>10	10–20
Blanco Serrano et al.(1999)	Spain	60	12	35
			18	27
			21	25
			25	22
			32	20
			40	17
			50	16
Correa et al.(2008)	Mexico	60	10	50
			20	40
			30	35
			40	30
			50	25
			60	20
Ding et al.(2015)	China	50	8.4	14
			10	20
			15	15
			25	10
			≥30	5–10
Gu et al.(2021)	China	4-12^a^	3.3	50
			10	50
			12.5	60
			20	50
			40	40
			60	40

^a^ Unbound valproic acid concentration.

## 4 Discussion

In this study, VPA PPK modeling in 103 Chinese pediatric patients with epilepsy was constructed, where clinical and genetic factors were investigated on the PK parameters of VPA. The final model showed satisfactory predictive performance and was used to facilitate the development of optimal dosing regimens for children. To the best of our knowledge, this is the first study to report that genetic polymorphisms of *ABCB1* have a significant effect on VPA CL/F in epileptic children.

The patient demographic characteristics, medication details and genotype were carefully documented in this analysis and used for reliable estimates of CL/F and its influential factors. During the modeling process (Table 3), the patient’s body weight and age were both found to have a significant effect on CL/F. However, the very close correlation in the children between weight and age (*r* = 0.915) requires the exclusion of one of these two variables. Age was included in the final regression model due to a greater impact on CL/F than weight.

Glucuronidation is a major pathway of VPA metabolism. The hepatic glucuronidation activities are low in infants, especially in children under 2 years old, and reach the adult levels after 10–15 years of age (Strassburg et al., 2002). VPA is metabolized more quickly in younger children, and declines gradually with age. The CL/F of VPA is similar to those of adults when the child’s weight reaches 40 kg which is the mean weight of 12-year-old children (Ogungbenro and Aarons, 2014; Methaneethorn, 2018). Thus, the age-dependent changes in the VPA CL/F of our study may partially be explained by the abundance of hepatic drug enzymes that changes significantly during growth (Kearns et al., 2003). An age-dependent exponent model was used by Ding et al. (2015) to identify the maturation processes of VPA CL/F. Furthermore, VPA is high protein binding with albumin (90%–95%). When the albumin content in the blood decreases, the CL/F value of VPA increases due to more unbound VPA (Kearns et al., 2003). The serum albumin concentration increases with age in children, suggesting CL/F of VPA decreases with increasing age (Doré et al., 2017). Albumin levels was reported to be a significant factor affecting VPA CL/F and a table of individualized medication regimens based on albumin levels was constructed by Guo et al. (2020).

Additionally, it is well known that weight is an important cause of PK variation among individuals and is related to the functionality and development of the organs responsible for drug elimination. Several studies have demonstrated that weight is an important factor influencing the PK process of VPA in children with epilepsy (Correa et al., 2008; Ding et al., 2015; Xu et al., 2018). Notwithstanding the wide usage of the 3/4 allometric exponent method to scale CL/F (Holford et al., 2013; Back et al., 2019), the value of 0.75 remains controversial due to over-predicting CL/F for neonates and under-predicting CL/F for infants (Peeters et al., 2010). Although body weight is known to be an important factor in dosing regimens for the pediatric patient, weight-based dosing may raise concerns of adverse effects in obese children (Löscher, 2002), and the effect of age should be considered as well.

Genetic variants had been proved to influence the pharmacokinetics of VPA and contribute to its IIV, however, only a few studies calculated the PPK parameters of VPA involving genetic polymorphisms as covariates. *CYP2C9* and *CYP2C19* genotypes were found to significantly affect VPA CL/F (Jiang et al., 2007; Guo et al., 2020). However, other studies indicated that none of the CYPs or UGTs gene variants affect the VPA PK (Xu et al., 2018). As metabolism by the CYP pathway is not the main route of VPA elimination, other genetic polymorphisms that might cause variations in VPA PK should be considered. CL/F in Chinese epileptic children with the *LEPR* rs1137101 variant (AG and GG genotypes) were much lower than in those with the AA genotype (17.8% and 22.6% lower, respectively) (Xu et al., 2018).

In the present study, 12 selected genes related to ABC transporters, VPA metabolism, nuclear receptors, and the efficacy of VPA treatment were included as covariates to evaluate their influence on VPA CL/F. CL/F in patients with the *ABCB1* rs3789243 GG and AG genotypes differed from those with the AA genotype (8% higher and 4.7% lower, respectively) through the PPK model development. *ABCB1* gene encodes the membrane-associated protein [P-glycoprotein (P-gp/*ABCB1*)], a member of the superfamily of ABC transporters, which limits the intracellular uptake and retention of various molecules. Membrane transporters are important determinants of drug absorption, distribution and elimination (Yee et al., 2010). ABC transporters have been reported to be associated with antiepileptic drug resistance (Kwan et al., 2009; Chen et al., 2018; Al-Eitan et al., 2019; Chouchi et al., 2019). As *ABCB1* genotypes could affect the disposition of VPA, this could explain why it leads to PK diversity of VPA between individuals. It is essential for future research to clearly clarify why heterozygous carriers and homozygous carriers showing functional consequences in two directions.

The *SCN1A* gene encodes the alpha 1 subunit of the voltage-gated Na+ channel and plays a crucial role in the pathogenesis of several epilepsy syndromes (Scheffer and Nabbout, 2019). *SCN1A* gene polymorphisms were found to be associated with the therapeutic effects of VPA in the treatment of epilepsy (Wang et al., 2018; Shi et al., 2019). The *SCN1A* gene polymorphisms were selected as covariates in our PPK analysis, but none of them were added to the final model.

Concomitant medications commonly found to influence VPA pharmacokinetic characteristics *in vivo* included carbamazepine, phenobarbitone and phenytoin due to their enzyme-inducing capacity (Methaneethorn, 2018). However, the effect of concomitant medications on VPA CL/F was not significant in this PPK modeling study, partly because of the limited number of subjects receiving these drugs concomitantly. In the forward inclusion, concomitant therapy with clonazepam reduced the OFV by 3.920 (*p* < 0.05), but the effect was removed from the final regression model. Yukawa et al. found that concomitant administration of clonazepam showed a 17.9% decrease in VPA CL/F among 250 Japanese patients aged 0.3–32.6 years with an unknown mechanism of interaction (Yukawa et al., 2003), but other studies have shown that VPA concentrations were not affected by clonazepam (Wang and Wang, 2002; Zang et al., 2022). Therefore, the interactions between VPA and comedications should be further investigated.

An increase in VPA CL/F with increasing VPA total daily dose (TDD) was reported by several studies in both adult and pediatric patients (Correa et al., 2008; Nakashima et al., 2015; Methaneethorn, 2018). This could be explained by the protein binding saturation, resulting in a higher unbound fraction of VPA concentrations available for elimination and therefore higher CL/F (Kodama et al., 1992). Moreover, the TDM effects should also be considered: patients with high CL/F tend to receive a higher dosage to ensure the concentration within the therapeutic range (Ahn et al., 2005). Therefore, the dose-dependent maximum effect (DDE) model and protein binding model were found to best describe VPA data (Ding et al., 2015). In our analysis, the effect of TDD on VPA CL/F was investigated to describe the characteristics of protein binding and then found to be not significant.

Whether sex affects the CL/F of VPA is controversial in previous studies. Some studies have reported that female patients have lower CL/F of VPA because of the difference in weight between males and females (Ibarra et al., 2013; Nakashima et al., 2015). UGT activity in females lower than in males could also account for the sex-induced differences in VPA CL/F (Court, 2010). However, this finding was confirmed neither by our study, nor by other VPA PPK models (Serrano et al., 1999; Correa et al., 2008; Williams et al., 2012; Ding et al., 2015). Whether the 1:1 male to female ratio affects VPA pharmacokinetic properties should be investigated in further studies in a larger sample size.

The simulations of VPA dosing (Table 5) based on the age and *ABCB1* genotype of patients indicated that 20–25 mg/kg/day bid of VPA oral administration is sufficient to maintain PTA >70% for most patients aged 1–10 years, while the same dose to patients with GG genotype would lead to relatively lower VPA concentrations. As shown in Figure 3, the Monte Carlo simulations evidenced that dose requirements decreased as weight increased for the typical weight of 10–40 kg in children. The model-based recommended doses are 20–30 mg/kg/day in the present study with higher doses such as 40 mg/kg/day for lower weight subgroups of 10–15 kg. Current recommendations indicated a maintenance daily dose of 20–30 mg/kg for children, which is consistent with our simulation results (Braathen et al., 1988; Rodrigues et al., 2018). A summary of the dosage regimens recommended by the other established PPK mode is displayed in Table 6. Dosage regimens based on our model are similar to Ding et al. in China and lower than that of western people (Correa et al., 2008; Ding et al., 2015) because of considering the high variability of VPA concentrations (50–100 mg/L *versus* around 60 mg/L) in our study. Based on the inter-individual and residual variability in the model, TDM is essential for guaranteeing VPA within the concentration targets and obtaining more accurate estimations with the Bayesian method as a basis for appropriate dosage adjustments.

Only a relatively small sample size was used in this study, which may result in non-significant differences on the included covariates. The predictive capacity of the final model was not evaluated by external validation, as data with genetic polymorphism could not be obtained from routine clinical information of patients. Moreover, actual clinical practice of the developed model and data on pharmacodynamics (PD) is lacking. Therefore, a further multicenter study with an increased sample size is needed to explore the PPK/PD model of VPA to determine the recommended therapeutic concentration for pediatric population with epilepsy and to verify the role of genetic factors on VPA PPK.

## 5 Conclusion

In this study, a novel PPK model enrolled Chinese pediatric patients with epilepsy for VPA was developed and proved to be stable with acceptable predictive performance. To the best of our knowledge, this study is the first to report that *ABCB1* genetic polymorphisms were identified as effective covariates for the CL/F of VPA. These findings contribute to a better understanding of the IIV in VPA PK, and a table of individualized medication regimens in consideration of the age and genotype was constructed to improve the therapeutic effect of VPA.

## Data Availability

The original contributions presented in the study are included in the article/supplementary material, further inquiries can be directed to the corresponding author.

## Abbreviations

VPA, Valproic acid; ILAE, International League against Epilepsy; AED, Antiepileptic drug; TDM, Therapeutic drug monitoring; PK, Pharmacokinetic; PPK, Population pharmacokinetic; LEPR, Leptin receptor; SCN1A, Sodium channel neuronal type I alpha subunit; NONMEM, Non-linear mixed-effects modeling; SNP, Single nucleotide polymorphism; OFV, Objective function value; AIC, Akaike information criterion; BIC, Bayesian information criterion; GOF, Goodness-of-fit; CWRES, Conditional weighted residual; VPC, Visual predictive checks; PTA, Probability of target attainment; RSE, Relative standard error; IIV, Inter-individual variability; RV, Residual variability; TDD, Total daily dose.
